# Risk Factors and Outcomes of Early Relapse After Curative Resection of Intrahepatic Cholangiocarcinoma

**DOI:** 10.3389/fonc.2019.00854

**Published:** 2019-09-04

**Authors:** Hua Yang, Jie Wang, Zehuan Li, Yi Yang, Liuxiao Yang, Yong Zhang, Yinghong Shi, Ya Cao, Jian Zhou, Zheng Wang, Qing Chen

**Affiliations:** ^1^Department of General Surgery, Shanghai Public Health Clinical Center, Zhongshan Hospital (South), Fudan University, Shanghai, China; ^2^ENT Institute and Otorhinolaryngology Department, Eye & ENT Hospital, Fudan University, Shanghai, China; ^3^Department of General Surgery, Zhongshan Hospital, Fudan University, Shanghai, China; ^4^Key Laboratory of Carcinogenesis and Cancer Invasion, Liver Cancer Institute, Zhongshan Hospital, Fudan University, Ministry of Education, Shanghai, China; ^5^Key Laboratory of Carcinogenesis and Cancer Invasion, Cancer Research Institute, Central South University, Ministry of Education, Changsha, China; ^6^Institute of Biomedical Sciences, Fudan University, Shanghai, China; ^7^State key Laboratory of Genetic Engineering, Fudan University, Shanghai, China

**Keywords:** intrahepatic cholangiocarcinoma, early relapse, CA19-9, liver resection, prognosis

## Abstract

Early relapse after hepatectomy for intrahepatic cholangiocarcinoma (ICC) has a tremendous influence on the long-term survival outcomes of ICC patients. The purpose of our study was to investigate risk factors for early tumor relapse and confirm whether early relapse was correlated with ICC patients' long-term survival outcomes. Three hundred and twenty-two consecutive ICC patients undergoing partial hepatectomy at Liver Surgery Department of Zhongshan Hospital (Fudan University, Shanghai, China) between January 2005 and December 2011 were included in this retrospectively study. The definition of early relapse had been described as tumor relapse within 24 months after hepatectomy in ICC patients. We identified a total of 168 ICC patients with early relapse and 23 ICC patients with late relapse after hepatectomy. From the time of relapse, the long-term survival outcomes were worse among patients who had early vs. late relapse (median OS 16.5 vs. 44.7 months, respectively; *P* < 0.0001). The overall survival of the early relapse group was lower than that of the late relapse group (*P* < 0.0001). Multivariate Cox regression analysis indicated that multiple tumors (hazard ratio [HR], 1.951; 95% CI, 1.382–2.755; *P* < 0.001), lymphonodus metastasis (HR, 1.517; 95% CI, 1.061–2.168; *P* = 0.022), and higher serum CA19-9 levels (HR, 1.495; 95% CI, 1.095–2.039; *P* = 0.011) were independent risk factors of early relapse. Moreover, multiple tumors (HR, 1.641; 95% CI, 1.120–2.406; *P* = 0.011), lymphonodus metastasis (HR, 2.008; 95% CI, 1.367–2.949; *P* < 0.001), elevated NLR (HR, 1.921; 95% CI, 1.331–2.774; *P* < 0.001) and higher serum CA19-9 levels (HR, 1.990; 95% CI, 1.409–2.812; *P* < 0.001) were independent predictors of overall survival for ICC patients with early relapse. Collectively, our findings demonstrated that multiple tumors, lymphonodus metastasis, and higher serum CA19-9 levels were associated with the increased risks of early relapse and worse prognoses of ICC after curative-intent resection.

## Introduction

Intrahepatic cholangiocarcinoma (ICC) is the most common primary liver cancers that have an incidence inferior only to hepatocellular carcinoma (HCC). It accounts for 10–15% of all primary hepatic malignancy (1). ICC is a relatively rare and lethal liver malignancy which often has more aggressive tumor behaviors than HCC (2). With the appropriate patients' selection, surgery is considered the mainstay of curative-intent treatment option for ICC (3–5). Unfortunately, the long-term prognosis following curative-intent liver resection for ICC patients remains disappointing because of the high risk of cancer relapse. The incidence of relapse is up to 60–70% in ICC patients within 5 years after hepatectomy (6–8). Identifying risk factors of early relapse are critical to improving long-term survival outcomes after curative resection of ICC.

Hepatocellular carcinoma (HCC) relapse could be divided into early relapse (≤24 months) and late relapse (>24 months) basing on the time to tumor relapse after partial hepatectomy (9–12). Previous studies suggested that early relapse after curative resection of HCC has been associated with certain tumor pathological characteristics (e.g., multiple tumors, satellite nodules, large tumor size, macroscopic, and microscopic vascular invasion, and poor cell differentiation) (13–19). However, there is a lack of effective assessment criteria of clinical features of ICC patients as high risk of early relapse after curative-intent liver resection.

To our knowledge, the risk factors, the patterns of tumor relapse, and long-term prognosis for ICC with early relapse after curative-intent liver resection have been poorly studied. The objective of the current study was to assess the risk factors and predictors of long-term prognosis in ICC patients with early relapse after partial hepatectomy. The findings indicated that ICC patients with a high risk of early relapse were strongly recommended for closely tumor surveillance. This improved the chance of ICC patients to undergo curative resection or adjuvant therapy, thus, contributing to better survival for these ICC patients.

## Methods

### Patient Selection

All patients underwent partial hepatectomy with curative intent for ICC at Liver Surgery Department of Zhongshan Hospital (Shanghai, China) from January 2005 to December 2011. A total of ICC 322 patients were enrolled in this retrospective study. This study was approved by the Zhongshan Hospital Ethics Committee and informed consent was obtained from each patient before surgery. Part of the physical examinations, including serum CA19-9, α-fetoprotein (AFP), and liver function test were performed within 1 week prior to surgery. ICC patients who underwent pre-operative therapy, such as transarterial chemoembolization (TACE), radiofrequency ablation (RFA), or percutaneous ethanol injection (PEI), were excluded from this study (20).

### Follow-Up Strategy

The patient's follow-up and post-operative management were carried out as described previously based on our established guidelines (21, 22). Briefly, all ICC patients were regularly followed up once every 2 months in the first 24 months after surgery and then every 3–6 months interval until death. Blood was taken for serum CA19-9, AFP, and liver function tests, as well as liver ultrasonography, were routinely carried out. Contrast-enhanced computerized tomography scanning (CT), or/and magnetic resonance imaging (MRI) was performed once every 6 months or more frequently when ICC relapses or metastasis was suspected. Further investigations, such as positron emission tomography CT (PET-CT) and hepatic angiography, were performed when clinically indicated. Overall survival (OS) was calculated from the interval between the dates of partial hepatectomy and death or between the dates of partial hepatectomy and the last observation. Time to relapse (TTR) was calculated from the interval between the dates of partial hepatectomy and first relapse or metastasis.

### Treatment of Relapse

When relapses of ICC were being confirmed, appropriate management included a second partial hepatectomy, TACE, RAF, external radiotherapy, or PEI were carried out based on the number of tumors, tumor diameter, tumor location of the recurrent tumors, general patient's condition, and liver function (21, 22).

### Statistical Analysis

Statistical analyses were assessed using SPSS 25.0 (IBM, New York, USA). Categorical variables were assessed using the χ^2^ test or Fisher's exact test, as appropriate. Continuous variables were assessed using Student's *t-*test or Mann-Whitney *U-*test. According to results from our previous studies (20, 22, 23), the optimal cutoff values for PLR, NLR, LMR, and CA19-9, which could be a potential association with prognosis, were selected to this study. The OS and cumulative relapse rates were analyzed using the Kaplan-Meier method and differences were compared using the log-rank test. Cox regression analyses were used for multivariate analyses. *P* < 0.05 was considered statistically significant for all analyses.

## Results

### The Occurrence of Early Relapse

**Figure 1:**
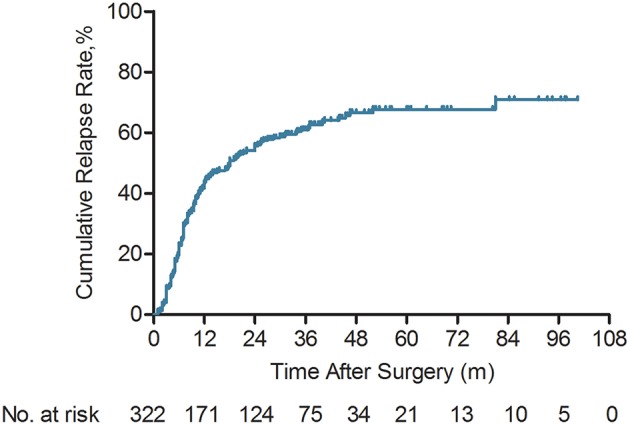
Kaplan-Meier analyses of cumulative relapse rate for intrahepatic cholangiocarcinoma (ICC) patients (*n* = 322).

A total of 322 ICC patients were enrolled in this retrospective study. The median follow-up time was 44.0 months (2.7–100.5 months). One hundred and eighty-seven (58.1%) ICC patients had died and tumor relapse occurred 191 (59.3%) patients at last follow-up. The cumulative 1-, 3-, and 5-year OS rates were 75.0, 47.8, and 35.2%, respectively. The cumulative relapse rates at the 1st, 3rd, and 5th year were 43.4, 61, and 67.7%, respectively (Figure 1). One hundred and sixty-eight of 322 ICC patients (52.2%) suffered cancer relapse within 2 years after surgery (early relapse), 23 (7.1%) after 24 months (late relapse), and 131 (40.7%) patients did not.

Comparisons of the clinicopathological parameters between the early and without tumor relapse of ICC patients were presented in Table 1. Among 168 ICC patients with early relapse, 104 (61.9%) were male and 64 (38.1%) were female; 67 patients (39.9%) had chronic HBV infection and one patient (0.6%) were positive for hepatitis C virus RNA. Compared with ICC patients without relapse, the patients with early relapse who had certain aggressive biological features, including multiple tumors (*P* = 0.017), lymphonodus metastasis (*P* = 0.001), microvascular invasion (*P* = 0.032), and higher serum CA19-9 levels (*P* = 0.009), as well as advanced TNM stage (*P* = 0.003).

**Table 1:** Clinical and pathological characteristics of patients with early and without relapse after curative liver resection for intrahepatic cholangiocarcinoma (*n* = 299).

**Clinicopathological indexes**		**No. (%)**			
		**Total (*N* = 299)**	**Early Relapse (*N* = 168)**	**Without Relapse (*N* = 131)**	***P-*value**
Age (year)	≤50	75 (25.1)	41 (24.4)	34 (26.0)	0.759
	>50	224 (74.9)	127 (75.6)	97 (74.0)	
Sex	Female	118 (39.5)	64 (38.1)	54 (41.2)	0.583
	Male	181 (60.5)	104 (61.9)	77 (58.8)	
HBsAg	Negative	178 (59.5)	101 (60.1)	77 (58.8)	0.815
	Positive	121 (40.5)	67 (39.9)	54 (41.2)	
HCV	Negative	297 (99.3)	167 (99.4)	130 (99.2)	1^*^
	Positive	2 (0.7)	1 (0.6)	1 (0.8)	
AFP (ng/ml)	≤20	264 (88.3)	150 (89.3)	114 (87.0)	0.546
	>20	35 (11.7)	18 (10.7)	17 (13.0)	
Child-Pugh	A	289 (96.7)	164 (97.6)	125 (95.4)	0.343
	B or C	10 (3.3)	4 (2.4)	6 (4.6)	
Liver cirrhosis	No	218 (72.9)	119 (70.8)	99 (75.6)	0.360
	Yes	81 (27.1)	49 (29.2)	32 (24.4)	
Tumor size (cm)	≤5	131 (43.8)	67 (39.9)	64 (48.9)	0.121
	>5	168 (56.2)	101 (60.1)	67 (51.1)	
Tumor number	Single	229 (76.6)	120 (71.4)	109 (83.2)	**0.017**
	Multiple	70 (23.4)	48 (28.6)	22 (16.8)	
Lymphonodus node metastasis	Yes	55 (18.4)	42 (25.0)	13 (10.0)	**0.001**
	No	244 (81.6)	126 (75.0)	118 (90.0)	
Microvascular invasion	Yes	42 (14.0)	30 (17.9)	12 (9.2)	**0.032**
	No	257 (86.0)	138 (82.1)	119 (90.8)	
Tumor differentiation^a^	Poor	66 (22.1)	36 (21.4)	30 (22.9)	0.550
	Moderated	184 (61.5)	101 (60.1)	83 (63.4)	
	Well	49 (16.4)	31 (18.5)	18 (13.7)	
TNM stage^b^	I + II	226 (75.6)	116 (69.0)	110 (84.0)	**0.003**
	III + IVA	73 (24.4)	52 (31.0)	21 (16.0)	
NLR	Low	135 (45.2)	70 (41.7)	65 (49.6)	0.170
	High	164 (54.8)	98 (58.3)	66 (50.4)	
PLR	Low	156 (52.2)	82 (48.8)	74 (56.5)	0.187
	High	143 (47.8)	86 (51.2)	57 (43.5)	
LMR	Low	195 (65.2)	115 (68.5)	80 (61.1)	0.184
	High	104 (34.8)	53 (31.5)	51 (38.9)	
CA19-9, U/ml	≤89	190 (63.5)	96 (57.1)	94 (71.8)	**0.009**
	>89	109 (36.5)	72 (42.9)	37 (28.2)	

*Boldface type indicates significant values*.

*AFP, alpha-fetoprotein; CA19-9, carbohydrate antigen 19-9; HBsAg, hepatitis B surface antigen; HCV, hepatitis C virus; PLR, platelet-to-lymphocyte ratio; LMR, lymphocyte-to-monocyte ratio; NLR, neutrophil-to-lymphocyte ratio; TNM, tumor-node-metastasis; P, poor differentiation; M, moderated differentiation; W, well-differentiation*.

a *Tumor differentiation was determined according to the “British Society of Gastroenterology guidelines on the management of cholangiocarcinoma”*.

b *TNM stage: American Joint Committee on Cancer 7th edition staging for intrahepatic cholangiocarcinoma*.

* *Fisher's exact tests; chi-square tests for all other analyses*.

**Figure 2:**
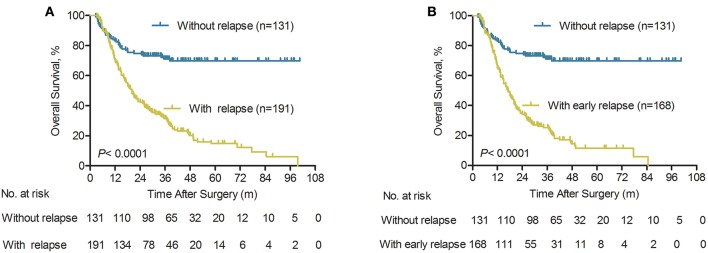
Overall survival of patients according to the type of relapse after initial surgery for ICC. **(A)** Overall survival curves of ICC patients with relapse and without relapse (*n* = 322), *P* < 0.0001 (log-rank test). **(B)** Overall survival curves of ICC patients with early relapse and without relapse (*n* = 299), *P* < 0.0001 (log-rank test).

ICC patients without post-operative relapse had better survival than those who developed ICC relapse (median OS 35.7 vs. 19.3 months). The cumulative 1-, 3-, and 5-year survival rates for ICC patients without relapse were 83.2, 71.1, and 69.8%, respectively, which were significantly higher compared with those of the relapse group (69.3, 31.5, and 14.8%, respectively; *P* < 0.0001, Figure 2A). Notably, ICC patients who had a tumor relapse within 24 months had a shorter median OS than those without postoperative tumor relapse (median OS 16.5 vs. 35.7 months). For ICC patients with early relapse, the cumulative 1-, 2-, and 3-year survival rates were 65.1, 25.4, and 11.6%, respectively. Which were significantly lower compared with the non- relapse group (83.2, 71.1, and 69.8%, respectively; *P* < 0.0001, Figure 2B).

### Comparison of Early and Late Relapse

Among the 322 ICC patients, 168 (52.2%) had a relapse within the first 2 years post-surgery (early relapse group) and 23 (7.1%) did over 2 years after surgery (late relapse group); without relapse group comprised 131 ICC patients. The overall survival of late relapse group was significantly prolonged, compared with the early relapse group (median OS 44.7 vs. 16.5 months). For the early relapse group, the OS rates for 1-, 3-, and 5-year were 65.1, 25.4, and 11.6%, respectively, and for the late relapse group were 100, 76.0, and 39.6% (*P* < 0.0001, Figure S1A). In addition, we further investigated the 168 early relapses after partial hepatectomy within the 24-month period in the analyzed cohort. The results for the OS rates of ICC patients in the 1- and 2-year relapse groups were shown in Figure S1B. Our findings revealed that for the 2-year relapse group, the 1-, 3-, and 5-year OS rates were100, 60.8, and 38.5%, respectively (Figure S1B). The long-term survival outcomes were significantly better compared with the 1-year relapse group who had rates of 56.1, 16.1, and 5.1% for 1-, 3-, and 5-year OS, respectively (*P* < 0.0001, Figure S1B).

### Risk Factors of ICC Patients With Early Relapse

Univariate analysis demonstrated that clinical features, including multiple tumors (*P* < 0.001), lymphonodus metastasis (*P* = 0.014) and elevated serum CA19-9 levels (*P* = 0.013), which could be correlated with an increased risk of early relapse within 2 years after curative resection of ICC (Table 2). Moreover, multivariate Cox regression analysis demonstrated that multiple tumors (HR, 1.951; 95% CI, 1.382–2.755; *P* < 0.001), lymphonodus metastasis (HR, 1.517; 95% CI, 1.061–2.168; *P* = 0.022), and higher serum CA19-9 levels (HR, 1.495; 95% CI, 1.095–2.039; *P* = 0.011) were independent risk factors of ICC patients with early relapse (Table 2).

**Table 2:** Univariate and multivariate cox regression analyses of time to early relapse in patients who were relapse at 2 years after resection with curative intent for intrahepatic cholangiocarcinoma (*n* = 168).

**Variable**	**Univariate**		**Multivariate**	
	**HR (95% CI)**	***P***	**HR (95% CI)**	***P***
Age, year (≤50 vs. >50)	1.299 (0.913–1.849)	0.147	NA	NA
Sex (female vs. male)	0.884 (0.647–1.208)	0.440	NA	NA
HBsAg (negative vs. positive)	0.805 (0.592–1.096)	0.168	NA	NA
HCV (negative vs. positive)	0.305 (0.074–1.248)	0.098	NA	NA
AFP, ng/ml (≤20 vs. >20)	1.490 (0.910–2.439)	0.113	NA	NA
Child-Pugh (A vs. B or C)	1.030 (0.381–2.785)	0.953	NA	NA
Liver cirrhosis (no vs. yes)	1.108 (0.794–1.547)	0.545	NA	NA
Tumor size, cm (≤5 vs. >5)	1.167 (0.856–1.591)	0.329	NA	NA
Tumor number (single vs. multiple)	1.986 (1.409–2.799)	**0.000**	1.951(1.382–2.755)	**0.000**
Lymphonodus node metastasis (no vs. yes)	1.558 (1.093–2.219)	**0.014**	1.517(1.061–2.168)	**0.022**
Microvascular invasion (no vs. yes)	1.593 (0.960–2.643)	0.072	NA	NA
Tumor differentiation^a^ (P vs. M,W)	1.107 (0.815–1.502)	0.516	NA	NA
TNM stage^b^ (I+II vs. III+IVA)	1.336 (0.962–1.856)	0.084	NA	NA
NLR (low vs. high)	1.318 (0.968–1.795)	0.080	NA	NA
PLR (low vs. high)	1.259 (0.927–1.710)	0.140	NA	NA
LMR (low vs. high)	0.812 (0.584–1.128)	0.214	NA	NA
CA19-9, U/ml (≤89 vs. >89)	1.478 (1.084–2.016)	**0.013**	1.495 (1.095–2.039)	**0.011**

*NA, not applicable*.

*Boldface type indicates significant values*.

*Analyses were conducted using univariate analysis or multivariate Cox proportional hazards regression*.

*AFP, alpha-fetoprotein; CA19-9, carbohydrate antigen 19-9; HBsAg, hepatitis B surface antigen; HCV, hepatitis C virus; PLR, platelet-to-lymphocyte ratio; LMR, lymphocyte-to-monocyte ratio; NLR, neutrophil-to-lymphocyte ratio; TNM, tumor-node-metastasis; CI, confidential interval; P, poor differentiation; M, moderated differentiation; W, well-differentiation*.

a *Tumor differentiation was determined according to the “British Society of Gastroenterology guidelines on the management of cholangiocarcinoma”*.

b *TNM stage: American Joint Committee on Cancer 7th edition staging for intrahepatic cholangiocarcinoma*.

### Risk Factors for Poor Prognoses of ICC Patients With Early Relapse

Results from our univariate analysis demonstrated that HCV infection (*P* = 0.041), multiple tumors (*P* = 0.032), lymphonodus metastasis (*P* < 0.001), advanced TNM stage (*P* < 0.001), elevated neutrophil-to-lymphocyte ratio (NLR, *P* < 0.001), lower lymphocyte-to-monocyte ratio (LMR, *P* = 0.010) and higher serum CA19-9 levels (>89 U/ml, *P* < 0.001) were prognostic factors of OS for ICC patients with early relapse after liver resection (Table 3). Moreover, multivariate Cox regression analysis demonstrated that multiple tumors (HR, 1.641; 95% CI, 1.120–2.406; *P* = 0.011), lymphonodus metastasis (HR, 2.008; 95% CI, 1.367–2.949; *P* < 0.001), elevated NLR levels (HR, 1.921; 95% CI, 1.331–2.774; *P* < 0.001) and higher serum CA19-9 levels (HR, 1.990; 95% CI, 1.409–2.812; *P* < 0.001) were independent predictors of OS for ICC patients with early relapse after liver resection (Table 3).

**Table 3:** Univariate and multivariate cox regression analyses of factors associated with overall survival in patients who were relapse at 2 years after resection with curative intent for intrahepatic cholangiocarcinoma (*n* = 168).

**Variable**	**Univariate**		**Multivariate**	
	**HR (95% CI)**	***P***	**HR (95% CI)**	***P***
Age, year (≤50 vs. >50)	1.069 (0.719–1.591)	0.741	NA	NA
Sex (female vs. male)	1.009 (0.711–1.431)	0.962	NA	NA
HBsAg (negative vs. positive)	1.100 (0.783–1.547)	0.582	NA	NA
HCV (negative vs. positive)	0.228 (0.055–0.941)	**0.041**	0.620(0.144–2.664)	0.520
AFP, ng/ml (≤20 vs. >20)	0.978 (0.560–1.708)	0.938	NA	NA
Child-Pugh (A vs. B or C)	1.532 (0.486–4.827)	0.466	NA	NA
Liver cirrhosis (no vs. yes)	0.908 (0.621–1.326)	0.616	NA	NA
Tumor size, cm (≤5 vs. >5)	1.258 (0.888–1.781)	0.197	NA	NA
Tumor number (single vs. multiple)	1.508 (1.036–2.194)	**0.032**	1.641(1.120–2.406)	**0.011**
Lymphonodus node metastasis (no vs. yes)	2.147 (1.469–3.138)	**0.000**	2.008(1.367–2.949)	**0.000**
Microvascular invasion (no vs. yes)	1.004 (0.634–1.588)	0.987	NA	NA
Tumor differentiation^a^ (P vs. M,W)	0.916 (0.650–1.293)	0.619	NA	NA
TNM stage^b^ (I+II vs. III+IVA)	1.974 (1.379–2.826)	**0.000**	0.912(0.441–1.885)	0.803
NLR (low vs. high)	1.904 (1.329–2.729)	**0.000**	1.921(1.331–2.774)	**0.000**
PLR (low vs. high)	1.384 (0.985–1.944)	0.061	NA	NA
LMR (low vs. high)	0.606 (0.414–0.885)	**0.010**	0.761(0.489–1.182)	0.224
CA19-9, U/ml (≤89 vs. >89)	1.926 (1.369–2.710)	**0.000**	1.990 (1.409–2.812)	**0.000**

*NA, not applicable*.

*Boldface type indicates significant values*.

*Analyses were conducted using univariate analysis or multivariate Cox proportional hazards regression*.

*AFP, alpha-fetoprotein; CA19-9, carbohydrate antigen 19-9; HBsAg, hepatitis B surface antigen; HCV, hepatitis C virus; PLR, platelet-to-lymphocyte ratio; LMR, lymphocyte-to-monocyte ratio; NLR, neutrophil-to-lymphocyte ratio; TNM, tumor-node-metastasis; CI, confidential interval; P, poor differentiation; M, moderated differentiation; W, well-differentiation*.

a *Tumor differentiation was determined according to the “British Society of Gastroenterology guidelines on the management of cholangiocarcinoma”*.

b *TNM stage: American Joint Committee on Cancer 7th edition staging for intrahepatic cholangiocarcinoma*.

### The Prognostic Significance of Systemic Inflammatory Response and Serum CA19-9 in ICC Patients With Early Relapse

**Figure 3:**
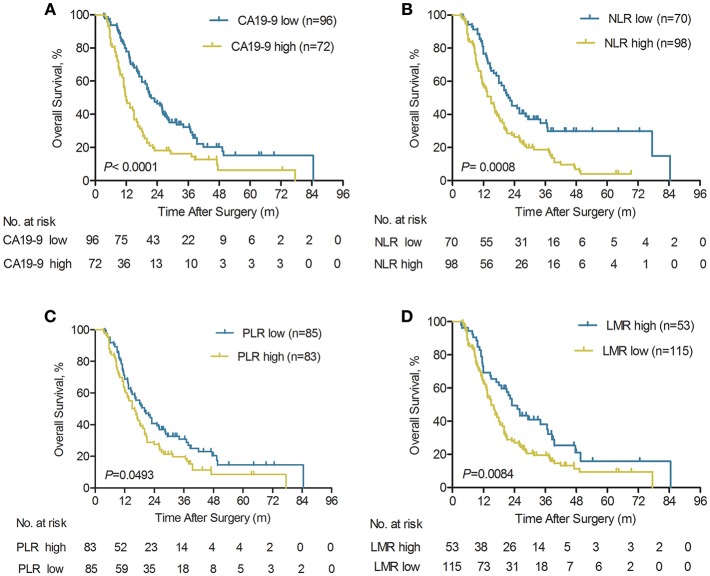
Kaplan-Meier analyses of overall survival rate for ICC patients with early relapse according to serum CA19-9, pre-operative NLR, PLR, and LMR. **(A)** Compared with the serum CA19-9 high group, OS was significantly higher in the serum CA19-9 low group (*n* = 168), *P* < 0.0001 (log-rank test). **(B)** Compared with the pre-operative NLR high group, OS was significantly higher in the NLR low group (*n* = 168), *P* = 0.0008 (log-rank test). **(C)** Compared with the pre-operative PLR high group, OS was significantly higher in the pre-operative PLR low group (*n* = 168), *P* = 0.0493 (log-rank test). **(D)** Compared with the pre-operative LMR low group, OS was significantly higher in the pre-operative LMR high group (*n* = 168), *P* = 0.0084 (log-rank test).

Our previous studies have shown that systemic inflammatory response, including tumor biomarker serum CA19-9, platelet-to-lymphocyte ratio (PLR), NLR, and LMR, had been proposed as prognostic biomarkers in ICC patients (20, 22, 23). In the current study, the prognostic value of the serum CA19-9, NLR, PLR, and LMR was further investigated in a large cohort of ICC patients with early relapse (*n* = 168). The Kaplan-Meier curves indicated that higher serum CA19-9, NLR, PLR, and lower LMR were all significantly associated with worse OS (all *P* < 0.05; Figures 3A–D). Our findings demonstrated that for the ICC patients with low CA19-9, the 1-, 3-, and 5-year OS rates were 77.9, 32.2, and 15.2%, respectively. The results were significantly better compared with the patients with elevated CA19-9 (≥89 U/ml) who had rates of 48.1, 16.3, and 6.3% for 1-, 3-, and 5-year OS, respectively (*P* < 0.0001, Figure 3A). In addition, the elevated NLR (≥2.49) and PLR (≥123) were significantly correlated with worse OS (*P* = 0.0008 and *P* = 0.0493, respectively; Figures 3B,C). Meanwhile, the higher LMR (>4.45) was also significantly associated with the prolonged OS (*P* = 0.0084; Figure 3D).

The results for cumulative relapse rates for ICC patients with early relapse according to systemic inflammatory response and serum CA19-9 levels were illustrated in Figure S2. The Kaplan-Meier curves indicated that higher serum CA19-9 (≥89 U/ml) and NLR (≥2.49) were both significantly associated with higher cumulative relapse rates (both *P* < 0.05, respectively; Figures S2A,B). The 6-, 12-, and 18-month cumulative relapse rates were significantly higher in the elevated CA19-9 group (52.8, 90.3, and 97.2%, respectively) compared with the low CA19-9 group (35.4, 71.9, and 88.5%, respectively; *P* = 0.0127, Figure S2A). Similarity, Cumulative relapse rates of high NLR group and low NLR group in ICC patients with early relapse were displayed in Figure S2B. The higher NLR was also significantly correlated with early relapse (*P* = 0.0496; Figure S2B). However, pre-operative PLR and LMR had no impact on the risk of early tumor relapse (Figures S2C,D). Thus, our findings demonstrated that ICC patients with elevated serum CA19-9 levels and NLR should be under regular relapse surveillance.

### The Prognostic Significance of Tumor Characteristics in ICC Patients With Early Relapse

**Figure 4:**
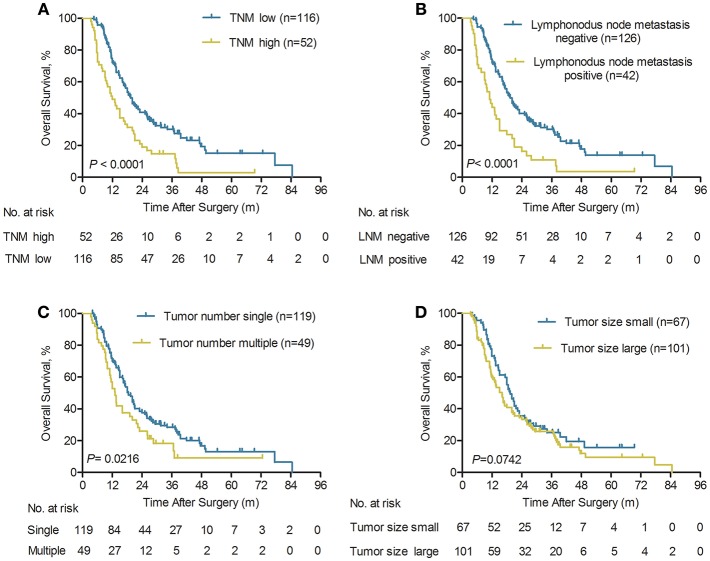
Kaplan-Meier analyses of overall survival rate for ICC patients with early relapse according to the TNM stage, lymphonodus node metastasis (LNM), tumor number, and tumor size. **(A)** Compared with the TNM high group, OS was significantly higher in the TNM low group (*n* = 168), *P* < 0.0001 (log-rank test). **(B)** Compared with the LNM positive group, OS was significantly higher in the LNM negative group (*n* = 168), *P* < 0.0001 (log-rank test). **(C)** Compared with the multiple tumors group, OS was significantly higher in the single tumor group (*n* = 168), *P* = 0.0216 (log-rank test). **(D)** The overall survival rate was no significant difference between small and large tumor size (*n* = 168), *P* = 0.0742 (log-rank test).

It was revealed in the Kaplan-Meier curves that advanced TNM stage, lymphonodus metastasis, and multiple tumors were all significantly correlated with worse OS (all *P* < 0.05, Figures 4A–C). Our findings revealed that the 1-, 3-, and 5-year OS rates in the TNM ^I+II^ patients with ICC were significantly better than the overall survival rates in the TNM ^III+IVA^ ICC patients (72.1 vs. 49.1%, 30.1 vs. 14.7%, and 15.1 vs. 2.9%, respectively; *P* < 0.0001, Figure 4A). Similarity, the long-term survival outcomes were more favorable in ICC patient without lymphonodus metastasis than with lymphonodus metastasis (median OS 19.3 vs. 11.0 months; *P* < 0.0001). Our findings revealed that the 1-, 3-, and 5-year OS rates in the ICC patients without lymphonodus metastasis were significantly higher than the long-term survival rates in the patients with lymphonodus metastasis (72.0 vs. 44.0%, 30.1 vs. 10.9%, and 13.9 vs. 3.6%, respectively; *P* < 0.0001, Figure 4B). In addition, the multiple tumors remained associated with shorter OS (*P* = 0.0216, Figure 4C). However, the long-term survival outcomes following curative treatment of ICC patients had no difference in small and large tumor size groups (median OS 19.3 vs. 16 months, *P* = 0.0742; Figure 4D).

The results for cumulative relapse rates for ICC patients with early relapse according to tumor characteristics were illustrated in Figure S3. The Kaplan-Meier curves indicated that advanced TNM stage, lymphonodus metastasis, and multiple tumors were all significantly correlated with higher cumulative relapse rates (all *P* < 0.05, Figures S3A–C). The 6-, 12-, and 18-month cumulative relapse rates were significantly higher in the advanced TNM stage group (59.6, 85.5, and 92.2%, respectively) compared with the low TNM stage group (35.3, 76.7, and 92.3%, respectively; *P* = 0.0174, Figure S3A). In addition, the cumulative relapse rates for lymphonodus metastasis and multiple tumors of early relapse in patients with ICC are shown in Figures S3B,C. Lymphonodus metastasis and multiple tumors were both significantly correlated with early relapse (both *P* < 0.05, Figures S3B,C). Meanwhile, tumor size has no impact on the risk of early tumor relapse (Figure S3D). Therefore, ICC patients with early relapse had certain aggressive biological features, including multiple tumors, lymphonodus metastasis, and advanced TNM stage were correlated with an increased likelihood of early relapse.

## Discussion

Intrahepatic cholangiocarcinoma (ICC) is one of the second common primary liver malignancies after HCC and the morbidity and mortality of ICC are increasing over recent decades worldwide (24, 25). Despite curative-intent surgical resection, the 5-year survival rate is still only 20–35% (7, 26). Unfortunately, the long-term prognosis for ICC after hepatectomy remains unsatisfactory, owing to the high risk of cancer relapse.

Hepatocellular carcinoma (HCC) patients with early vs. late relapse has been studied extensively (9, 11, 12). However, the topic on the risk factors, the patterns of tumor relapse, and long-term prognosis for ICC patient have been rarely studied. Based on the previous studies (8, 12), 2 years was determined as the best cutoff to distinguish early vs. late relapse of ICC patients. The present study demonstrated that more than half of patients [168 of 322 (52.2%)] with early relapse occurred at 2 years after resection with curative intent. Among these 168 patients with early relapse, 134 (79.8%) had a relapse in the first 12-month period and 34 (20.2%) with 24 months. In addition, the long-term survival outcomes of ICC patient with late relapse were more favorable, compared with ICC patient with early relapse. Moreover, ICC patients with early relapse had certain aggressive biological features, including multiple tumors, lymphonodus metastasis, the presence of microvascular invasion, and advanced TNM stage as well as higher serum CA19-9 levels.

Numerous previous studies have revealed that multiple tumors, large tumor diameters, poor cell differentiation, satellite nodules, as well as the presence of vascular invasion were independent risk factors of early relapse for HCC (13–19). However, there was a lack of reliable and effective evaluation criteria for high-risk features of early relapse in ICC patients after partial hepatectomy. In this retrospective study, our result showed that multiple tumors, lymphonodus metastasis, and elevated serum CA19-9 levels were independent risk factors of early relapse. The above findings were important as they strongly recommend that regular surveillance was intensely required during the first 24 months after partial hepatectomy of ICC, especially among ICC patients with aggressive biological features (e.g., multiple tumors, lymphonodus metastasis, and higher serum CA19-9 levels) who had been recommended to have potentially curative treatment or adjuvant therapy when early relapse was confirmed, so as to promote favorable prognoses for these ICC patients.

Interestingly, controversies exist on multiple tumors and tumor size as prognostic factors of ICC patients after partial hepatectomy. Yamasaki (27) proposed that ICC patients with a tumor diameter ≥ 2 cm had a worse prognosis. ICC patients with solitary or multiple tumors with a diameter of no more than 5 cm had a favorable prognosis, according to the sixth edition of the American Joint Committee on Cancer (AJCC) TNM staging system. However, Okabayashi et al. (28) reported that tumor size was not an independent risk factor for long-term survival. Our finding indicated that tumor diameter was not associated with the prognoses of ICC patients with early relapse after curative resection, consistent with the seventh edition of the AJCC TNM staging system. Similarity, our finding revealed that the long-term survival outcomes of ICC patient without lymphonodus metastasis were more favorable, compared with ICC with lymphonodus metastasis. Moreover, lymphonodus metastasis was an independent predictor of long-term survival for ICC patients with early relapse after partial hepatectomy. Lymphonodus metastasis reflected the aggressive biological features of ICC, and it was significantly correlated with worse prognoses of ICC patients. Thus, ICC patients with lymphonodus metastasis were recommended to undergoing postoperative radiotherapy for prevention of ICC relapse.

Inflammation has emerged as the seventh hallmark of cancer (29). Numerous previous studies have indicated elevated NLR was correlated with worse prognoses in multiple malignancies, such as colorectal carcinoma (30), non-small cell lung cancer (31), pancreatic carcinoma (32), and HCC (33, 34). Our previous study reported that ICC patients with elevated NLR suffered poor survival rates after partial hepatectomy (23). In the current study, our findings revealed that higher NLR was associated with a 1.92-fold increased mortality risk for ICC patients with early relapse in a multivariable analysis. Our previous findings confirmed that ICC cells recruit more tumor-associated neutrophils (TANs) to the tumor microenvironment, by secreting chemokines CXCL5. This process established a cancer-associated microenvironment, participates in the production of inflammatory mediators, amplifies the inflammatory response, and promotes tumor relapse and metastasis (35). Similarly, our recent study demonstrated that TANs recruit T regulatory cells (Tregs) and monocytes and into the cancer-associated microenvironment and differentiate into tumor-associated macrophages (TAMs), which facilitates the pro-metastasis niche formation, tumor-derived immunosuppression, tumor angiogenesis, relapse, and metastasis of HCC, as well as resistance to Sorafenib (36). Future studies should be conducted to determine whether tumor-associated inflammatory cells, including TAMs, TANs, and Tregs, affect the sensitivity of immunomodulation therapy in ICC patients.

Carbohydrate antigen 19-9 (CA19-9) was a readily accessible serum biomarker. Elevated serums CA19-9 had been used primarily in patients with pancreatic cancer, mixed HCC-cholangiocarcinoma, and extrahepatic cholangiocarcinoma (37–40). Previously, we had reported that the best cutoff value of serum CA19-9 ≥ 89 U/ml, higher serum CA19-9 level (≥89 U/ml) was an independent prognostic factor for ICC patients (22). However, the prognostic utility of serum CA19-9 has been rarely studied in ICC patients with early relapse after liver resection. In the present study, our findings indicated that higher serum CA19-9 levels were an independent risk factor of early relapse and mortality for ICC. In addition, our previous publication indicated that higher serum CA19-9 levels were significantly associated with large tumor diameters, lymphonodus metastasis, and advanced TNM stage (22). Furthermore, univariate and multivariate Cox regression analysis identified that higher serum CA19-9 levels were associated with 2.0-fold increased mortality risk for ICC patients with early relapse. Thus, ICC patients with elevated serum CA19-9 should be under intense postoperative relapse surveillance. This may improve the chance of ICC patients undergoing second partial hepatectomy and treat with further adjuvant therapy (TACE, RAF, and external radiotherapy).

## Limitations

This present study had several limitations. First, the study was a retrospective analysis, which may have selection biases. Second, as a single-center study, involved only patients from China, it was under-represented. Third, the majority of ICC patients [*n* = 67, (39.9%)] in China had a background of chronic HBV infection, whereas one patient [*n* = 1, (0.6%)] had anti-HCV positivity in the early relapse cohort (*n* = 168). However, primary sclerosing cholangitis and HCV infection were the key detrimental etiological factors for ICC in the United States, Europe, and Japan (22, 41, 42). In addition, the previous meta-analysis indicated HBV infection was associated with an increased risk of CCA in Asia (43). Moreover, the recent study revealed that antiviral therapy prolonged long-term survival for ICC patients with HBV-infected and a high viral level, by reducing viral reactivation (44). Therefore, our findings required large-scale prospective studies to further validate and make the results more convincing.

## Conclusions

In conclusion, the findings from our investigation demonstrated that several aggressive tumor characteristics (e.g., higher serum CA19-9 levels, multiple tumors, and lymphonodus metastasis) were significantly associated with increased risk factors of early relapse after curative liver resection for ICC. In addition, ICC patients who experienced early relapse had more unfavorable long-term survival outcomes than those with late relapse or without relapse. Moreover, elevated NLR, multiple tumors, lymphonodus metastasis, and higher serum CA19-9 levels predict poor clinical outcomes for ICC patients with early relapse. Therefore, closely tumor surveillance was strongly recommended for ICC patients with tumor-related risk factors. Further studies should be done to externally validate these biomarkers' ability to stratify risk among ICC patients with early relapse after surgical resection and to clarify the mechanisms for this possible relationship.

## Data Availability

The datasets generated for this study are available on request to the corresponding author.

## Author Contributions

QC and ZW: conception/design. HY, JW, ZL, YY, LY, YZ, YS, and YC: provision of study material or patients. QC, JW, ZL, YY, and LY: collection and/or assembly of data. QC, ZW, HY, JW, LY, and YY: data analysis and interpretation. QC, HY, JW, ZL, and YY: manuscript writing. HY, JW, ZL, YY, LY, YZ, YS, YC, JZ, ZW, and QC: final approval of manuscript.

### Conflict of Interest Statement

The authors declare that the research was conducted in the absence of any commercial or financial relationships that could be construed as a potential conflict of interest.

## Supplementary Material

Figure S1 Kaplan-Meier analyses of overall survival rate for ICC patients according to the type of relapse. **(A)** Overall survival curves of ICC patients with early and late relapse (*n* = 191), *P* < 0.0001 (log-rank test). **(B)** Overall survival curves of ICC patients with 1-year and 2-year relapse (*n* = 168), *P* < 0.0001 (log-rank test).

Figure S2 Kaplan-Meier analyses of cumulative relapse rate for ICC patients with early relapse according to serum CA19-9, pre-operative NLR, PLR, and LMR. **(A)** Compared with the serum CA19-9 ^high^ group, TTR (Time to recurrence) were significantly higher in the serum CA19-9 ^low^ group (*n* = 168), *P* = 0.0127 (log-rank test). **(B)** Compared with the pre-operative NLR ^high^ group, TTR was significantly higher in the pre-operative NLR ^low^ group (*n* = 168), *P* = 0.0496 (log-rank test). **(C)** Pre-operative PLR has no impact on the risk of early tumor relapse (*n* = 168), *P* = 0.1512 (log-rank test). **(D)** Pre-operative LMR has no impact on the risk of early tumor relapse (*n* = 168), *P* = 0.4730 (log-rank test).

Figure S3 Kaplan-Meier analyses of cumulative relapse rate for ICC patients with early relapse according to TNM stage, lymphonodus node metastasis (LNM), tumor number, and tumor size. **(A)** Compared with the TNM ^high^ group, TTR was significantly higher in the TNM ^low^ group (*n* = 168), *P* = 0.0174 (log-rank test). **(B)**. Compared with the LNM ^positive^ group, TTR was significantly higher in the LNM ^negative^ group (*n* = 168), *P* = 0.0063 (log-rank test). **(C)** Compared with the multiple tumors group, TTR was significantly higher in the single tumor group (*n* = 168), *P* < 0.0001 (log-rank test). **(D)** Tumor size has no impact on the risk of early tumor relapse (*n* = 168), *P* = 0.1039 (log-rank test).

## Abbreviations

AFP: α-fetoprotein
LMR: lymphocyte-to-monocyte ratio
CA19-9: carbohydrate antigen 19-9
HCC: hepatocellular carcinoma
HBV: hepatitis B virus
HCV: hepatitis C virus
ICC: intrahepatic cholangiocarcinoma
OS: Overall survival
PLR: platelet-to-lymphocyte ratio
TNM: tumor-node-metastasis
TTR: time to relapse
NLR: neutrophil-to-lymphocyte ratio.
