# Supplementary figures and images for: Risk Factors and Outcomes of Early Relapse After Curative Resection of Intrahepatic Cholangiocarcinoma
Source: Front Oncol. 2019 Sep 4;9:854. doi: 10.3389/fonc.2019.00854 (PMC6737003; doi:10.3389/fonc.2019.00854)

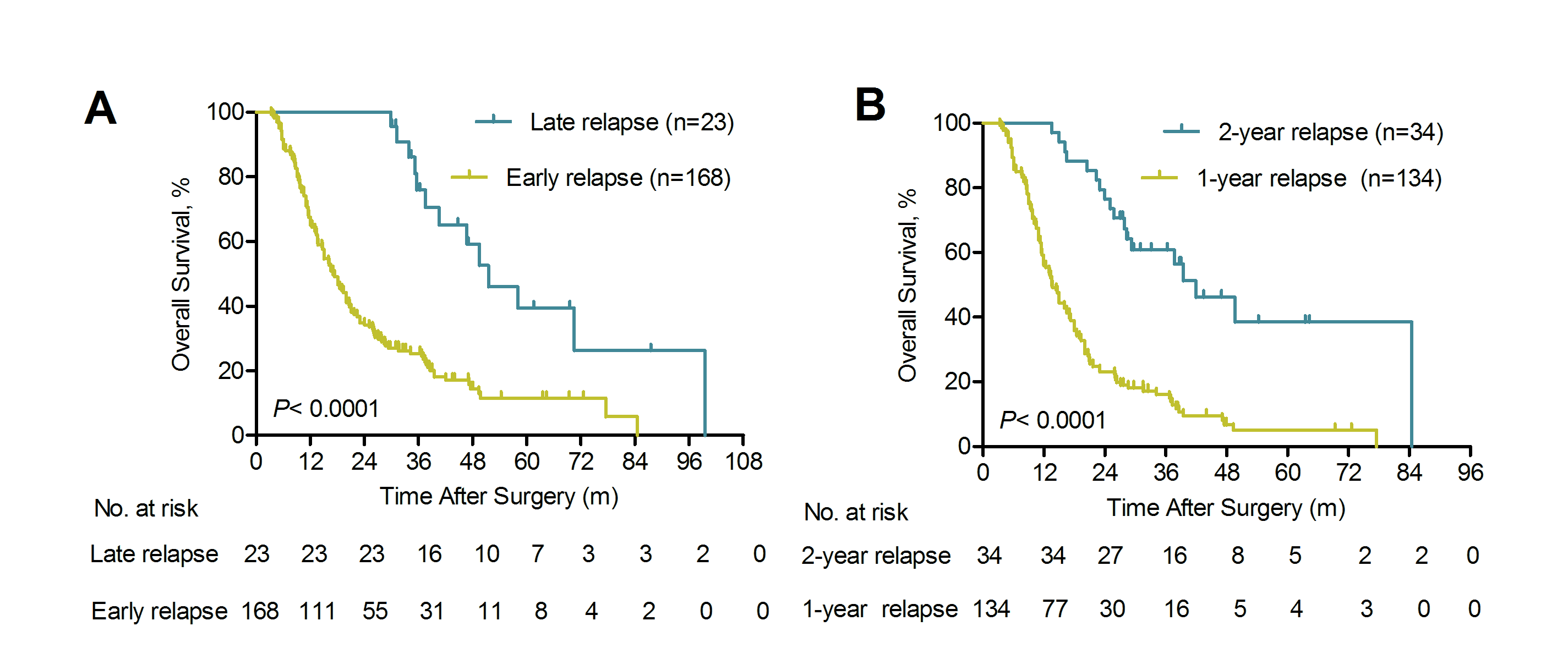

Supplement: Figure S1 — Kaplan-Meier analyses of overall survival rate for ICC patients according to the type of relapse. (A) Overall survival curves of ICC patients with early and late relapse (n = 191), P < 0.0001 (log-rank test). (B) Overall survival curves of ICC patients with 1-year and 2-year relapse (n = 168), P < 0.0001 (log-rank test). [file Image_1.TIF]

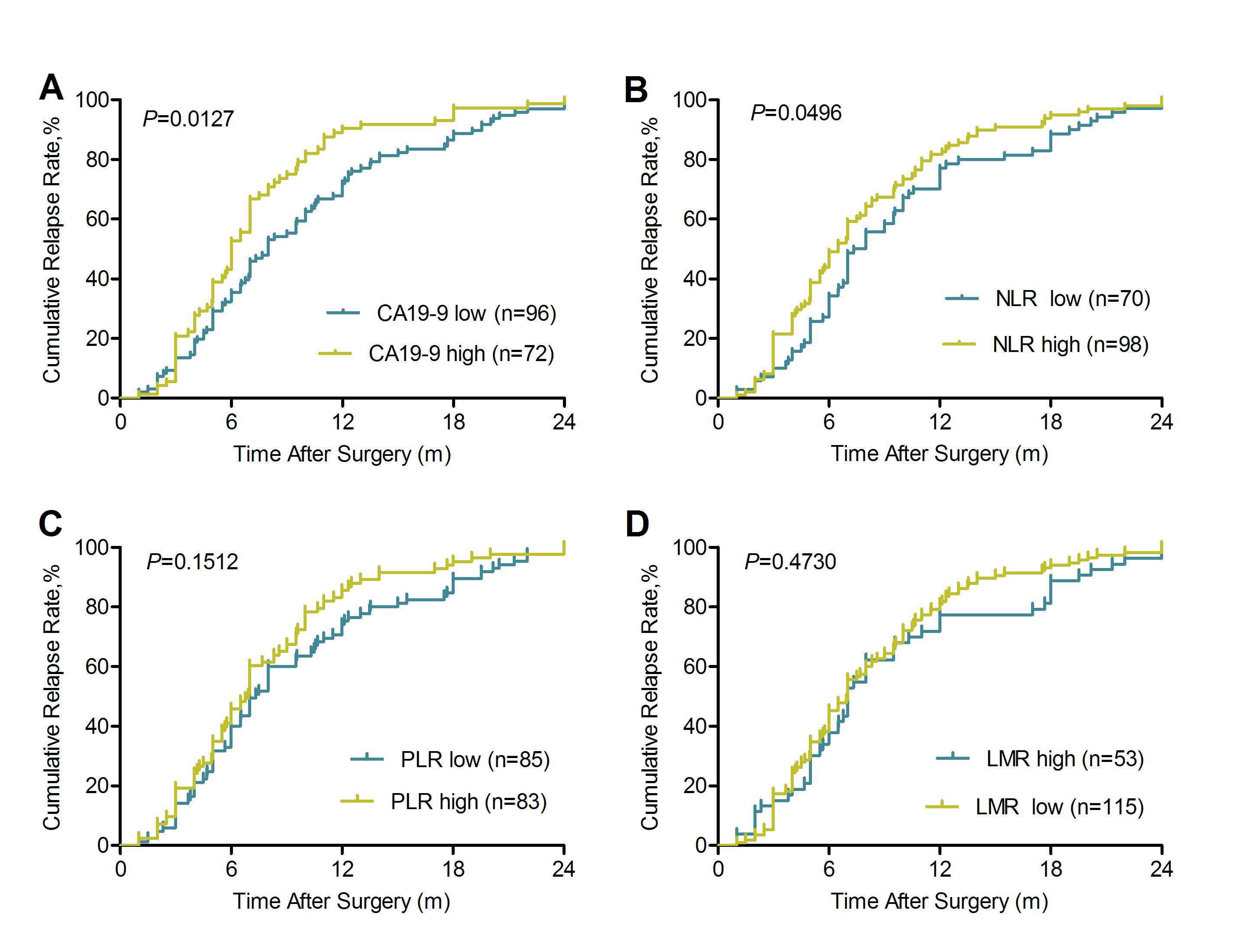

Supplement: Figure S2 — Kaplan-Meier analyses of cumulative relapse rate for ICC patients with early relapse according to serum CA19-9, pre-operative NLR, PLR, and LMR. (A) Compared with the serum CA19-9 high group, TTR (Time to recurrence) were significantly higher in the serum CA19-9 low group (n = 168), P = 0.0127 (log-rank test). (B) Compared with the pre-operative NLR high group, TTR was significantly higher in the pre-operative NLR low group (n = 168), P = 0.0496 (log-rank test). (C) Pre-operative PLR has no impact on the risk of early tumor relapse (n = 168), P = 0.1512 (log-rank test). (D) Pre-operative LMR has no impact on the risk of early tumor relapse (n = 168), P = 0.4730 (log-rank test). [file Image_2.TIF]

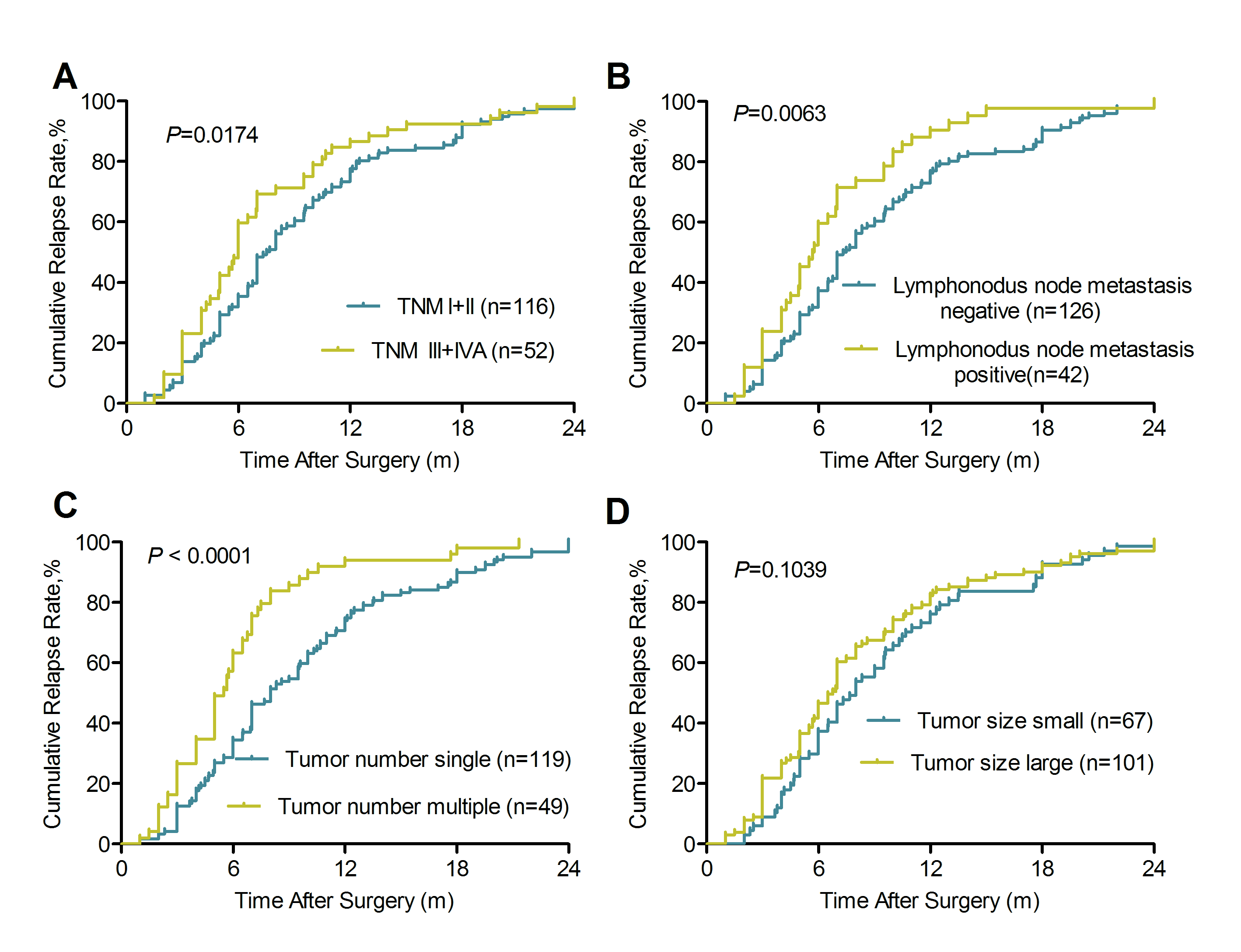

Supplement: Figure S3 — Kaplan-Meier analyses of cumulative relapse rate for ICC patients with early relapse according to TNM stage, lymphonodus node metastasis (LNM), tumor number, and tumor size. (A) Compared with the TNM high group, TTR was significantly higher in the TNM low group (n = 168), P = 0.0174 (log-rank test). (B). Compared with the LNM positive group, TTR was significantly higher in the LNM negative group (n = 168), P = 0.0063 (log-rank test). (C) Compared with the multiple tumors group, TTR was significantly higher in the single tumor group (n = 168), P < 0.0001 (log-rank test). (D) Tumor size has no impact on the risk of early tumor relapse (n = 168), P = 0.1039 (log-rank test). [file Image_3.TIF]
